# Neo-tropical species production: a sustainable strategy for climate change adaptation in neo-tropical regions

**DOI:** 10.1186/s12917-025-04558-6

**Published:** 2025-03-03

**Authors:** Laura Tardieu, Marc A. Driscoll, Kegan R. Jones

**Affiliations:** 1 Department of Food Production, Faculty of Food and Agriculture, The University of the West Indies, St. Augustine Campus, Eastern Main Road, St. Augustine, West Indies Trinidad and Tobago; 2 Department of Clinical Veterinary Sciences, School of Veterinary Medicine, Faculty of Medical Sciences, The University of the West Indies, St. Augustine Campus, Eastern Main Road, St. Augustine, West Indies Trinidad and Tobago; 3 Department of Basic Veterinary Sciences, School of Veterinary Medicine, Faculty of Medical Sciences, The University of the West Indies, St. Augustine Campus, Eastern Main Road, St. Augustine, West Indies Trinidad and Tobago

**Keywords:** Animal production, Climate change, Neo-tropical species, Wildlife

## Abstract

This opinion piece clarifies the impact of climate change on animal production in the Latin America and Caribbean (LAC) region and proposes a sustainable solution. Anthropogenic climate change has resulted in higher ambient temperatures, rainfall, humidity, storms and desertification. These events have direct and indirect effects on conventional animal performance and this piece will highlight the impact of increased temperatures on their welfare, health and production in the LAC. Alternative species such as neo-tropical wildlife animals have been proposed as climate resilient animals for use in the LAC, as they are well adapted to the climate and environment in the tropics. Some of these animals include capybara, lappe, agouti, caiman, cocrico and collared peccary. Neo-tropical animal production has the potential to produce nutritious meat, quality leather, reduce pollution and serve as a form of sustainable production. These animals can be inserted into a sustainable production system as their feed resources can be supplied through the use of local feedstuff, they also require less water and energy for maintenance, as they are well adapted to the high temperature and humidity in comparison to domesticated animals such as cattle, pigs and chickens. Finally, the key challenges including the legal use of the animals throughout the year, lack of technical experience and limited knowledge on the biology of these animals are discussed.

## Introduction

Climate change, primarily driven by anthropogenic activities, is significantly impacting many tropical and neo-tropical ecosystems [1]. This phenomenon can have negative effects, especially in developing regions like Latin America and the Caribbean (LAC). These negative effects can range from; increased mean environmental temperatures, with research citing up to a 4.5 °C increase across the LAC compared to pre industrial temperatures [2]; increasing aridity [3]; increased drought stresses due to wind pattern changes and the intertropical convergence zone (ITCZ) affecting precipitation and rainfall [4]; long term reduction in terrestrial water storage [5] and increased intensity of tropical cyclones, tropical storms, and hurricanes [2].

Impacts from climate change and negative environmental effects in the LAC are felt all the more keenly by agricultural ecosystems [6–8]. Agriculture is an important sector for LAC economies, employing a significant portion of the region’s workforce [9, 10]. Climate change, particularly the increase in temperatures, has been noted as negatively impacting the health and welfare of livestock species being utilized and produced [11, 12]. Livestock species often fall under traditional domesticated categories, including cattle (*Bos taurus)*, sheep (*Ovis aries*), goats (*Capra hircus)*, pigs (*Sus domesticus*), and poultry (*Gallus domesticus*). These species have, for the most part, evolved and been selectively bred primarily for temperate areas and within the environmental conditions that predominate cooler regions. Multiple reviews have been conducted on traditional livestock species revealing the stresses that climate change is having on animal productivity, health, and welfare [2, 8, 11, 12]. Heat stress, in particular has been identified as being responsible for reduced productivity, reduced animal welfare, decreased fertility, increased levels of disease susceptibility, and rises in mortality [13].

Research has focussed mainly on methods or strategies to alleviate the impacts of climate change on domesticated species. These studies range from shifting to better adapted [14] or smaller domesticated species [15]; improving housing [16]; enhancing nutrition or nutritional management [17, 18]; improving breeds [19–22]; digitalizing agriculture [9]; and investing in technology [22, 23]. Although many have proven promising, these strategies may not be adequate for the long-term climate changes that these regions are predicted to be facing, largely due to the costs and knowledge gaps that are associated with these proposed solutions [11]. Given the greater output required to feed the growing population and meet food security needs in the LAC region [8], more innovative solutions must be proposed.

One such solution, that can support domesticated animal production, involves the production and utilization of overlooked, indigenous animal resources that are found within the LAC region [24–29]. The main issue with the implementation of indigenous animal resources is the lack of knowledge of specific animal species as well as the dissemination of knowledge to local farmers to encourage these animals to be farmed in large numbers. This paper will focus on non-traditional and non-domesticated wildlife species, termed unconventional livestock in 1987 by Peters [30] or micro livestock by National Research Council in 1991 [31], that can prove to be both sustainable and climate smart solutions. Non-domesticated species that have been, or are being used or investigated for production in the LAC include the capybara (*Hydrochoerus hydrochaeris*) [32], collared peccary (*Pecari tajacu*) [33], spectacled caiman (*Caiman crocodilus*) [34, 35]; agouti (*Dasyprocta leporina*) [36, 37] green iguana (*Iguana iguana*) [38, 39], cocrico (*Ortalis ruficauda*) [123, 129] and other species.

Though novel, these species have already been adopted for use in the region. Given their natural evolutionary history and adaptation to the climate, endemic neo-tropical species have demonstrated a notable resilience to climate variability and environmental extremes. This resilience allows them to better withstand the impacts of climate change and to maintain their health more effectively [25] compared to traditional species [25]. Nogueira-Filho and da Cunha Nogueira (2000) have found that in comparison to domesticated species, wild, neo-tropical species like the capybara and collared peccary were able to thrive in their native neo-tropical habitat, particularly for meat production. Capybaras were found to display heat tolerance and local disease, and parasite resistance combined with overall hardiness and tolerance for poor nutrition [40].

Recent research on wild tropical birds provides additional support, showing that tropical birds including high-elevation bird species, possess the physiological capacities to deal with fluctuating temperatures [41]. In addition, tropical birds are equipped to endure elevated levels of heat, even in hot and arid environments [41]. These abilities indicate that tropical birds would be much better adapted than domesticated poultry breeds to the increased heat impacts of climate change.

This paper proposes that sustainable farming of endemic, neo-tropical, non-traditional species for the LAC region is a strategic approach to mitigate the adverse effects of climate change on animal health and welfare. This paper will further underscore the underutilization of local animal resources and the challenges associated with utilization of these species in the region. It will propose that developing production systems using these species is a sustainable option for improving food security in the Caribbean and Latin America, particularly in the context of climate change and animal health.

### Impacts of climate change on traditional domesticated species

Climate change is having a marked impact on livestock species globally. Research has identified various negative impacts of climate change on livestock production ranging from; decreased milk yields and milk quality, decreased meat production, to declines in fertility [42–44]. With the negative impacts of climate change, livestock-based food security, particularly for the LAC, is under threat [45].

Although animals can adapt to climatic environmental stresses, the increased heat stress negatively impacts livestock productivity by directly lowering animals’ adaptive response mechanisms, which can increase the spread and prevalence of diseases [43, 44, 46], lead to welfare issues [12, 47]; and indirectly impact the availability of feed crops and quality of forages [48].

Presently, climate change has negative effects on the productive parameters of domesticated species, impacting animal welfare, health and economic output. There have been extensive and diverse reviews that have highlighted and assessed these impacts (both directly and indirectly) on livestock production and food supply globally [13, 43, 44, 46, 49] and in developing countries [45] and this paper will not go into detail on this subject as it has already been detailed intensively. However, it is worth being noted that the impact of climate change on welfare, health and production of livestock species in the LAC region has been understudied particularly in the Caribbean area [50].

This paper will highlight the impact of heat stress and other impacts on the health of traditional livestock species and production with a focus on the LAC and the tropics, where possible.

### Heat stress

Heat Stress is primarily caused by high temperatures and high relative humidity; these climatic factors reduce the ability of animals to shed heat effectively to their environment. The effect of heat stress can vary with the species, breed, life stage, nutritional status, size and insulation level. Breeds and species with the highest energy demands are often the most susceptible [51]. Polsky and von Keyserlingk [12] examined the effects of heat stress on high producing dairy cattle and found that amongst cow breeds, *Bos taurus* breeds have been found to be more vulnerable than the more tropical breed *Bos indicus* to heat stress. This raises the concern about welfare and ethics, when animals that are not biologically adapted to a region’s climate and environment are introduced and bred under these conditions that result in poorer performance and increased illness, due to the pressures inherent in animal production [12]. The outcomes that arise in impacted specimens of heat stress on traditional livestock are often varied [42] with reduced feed intake being a symptom that arises, leading to declines in production rates and a reduction in animal welfare [12, 43, 52],.

Animal fertility is also affected [44, 53], and animals become increasingly vulnerable to diseases [43]. Heat stress may reduce the functioning of layer chickens’ immune system [54] and may also negatively affect the efficacy of vaccines in dairy cattle and broiler chickens [55, 56]. Studies also indicate that pastured livestock, like cattle, might become more vulnerable to certain plant poisons like ergot alkaloids that thrive in pasture grasses, leading to negative impacts on health, performance, and carcass traits [57, 58].

Dairy cattle and pig research have found that heat stress can affect and lower production. For example, dairy cattle were identified as having reduced milk produced in the first lactation impacting both the overall performance and health of dairy calves [12, 59]. Additionally, research conducted on pigs evaluating the impact of *in utero* heat stress on the performance of young born under these conditions, found that reducing heat stress on gestating pigs would likely result in improved pork yield and quality [60]. Conversely, for dairy cows exposed to heat stress while *in utero*, it was found that the young born from these cows are better adapted, with greater thermal tolerance to the heat and an increased ability to regulate their core temperatures [61, 62]. Declines in animal product quality have also been reported, with eggs displaying size and shell thickness reductions [54],, reductions in sheep milk yield [63] and reduced fat protein content observed in dairy cattle [64].

Meat products are also negatively affected with changes in colour, water-holding capacity, overall reduced shelf life, and food safety due to bacterial growth and shedding, associated with heat stress [65]. Consequently, with reductions in quality, food appeal of livestock products is altered, possibly leading to wastage increases and reduced prices. Ultimately, this leads to producers receiving reduced prices for their products and impacting their livelihoods. As mentioned above, although *B. indicus* has been found to be better adapted to the tropical conditions, *B. indicus* meat quality has been described as less tender [66, 67] and therefore less appealing. Thus, the selection of better-adapted climate-resilient domesticated breeds for the tropics may then equate to lower prices for farmers due to the lower consumer favourability.

Finally, reduction in feed intake, a symptom of heat stress, can act as a stressor and lead to the increased dissemination via shedding of pathogens such as *Escherichia coli* and Campylobacter bacteria, which can then be transferred from livestock to humans causing a health concern [68].

### Other impacts

Environmental impacts caused by climate change, including increased frequency and intensity of fires, droughts, storms and floods, can lead to loss of livestock [45, 69]. Infectious diseases alter overall disease susceptibility, which is intensified by changes in annual and seasonal cycles, impacting the incidence rate and severity of illness in livestock [55, 69]. This is as a result of the pathogens and hosts of parasites and insect vectors being sensitive to changes in climate, particularly humidity, temperature, and rainfall patterns [45, 69]. Often these diseases prove to be capable of transmission not only amongst livestock but also to humans [13, 45, 70].

Pinto et al. used projections and forecasts to examine climate change impacts on health with respect to animal diseases in South America [78]. This research concluded that climate change and disease spread are connected and influence each other through various mechanisms, and further that vector borne diseases and infectious animal disease distribution will likely change due to climate change in South America [78].

Studies have recently [71, 72] confirmed that dengue was the disease most reported across the largest number of climate types in the LAC. Concurrently, a review by Ashraf et al. [73] suggested that climate change resulted in the emergence and re-emergence of a variety of many zoonotic infectious diseases.

The increase in zoonotic diseases in the tropics is often partially attributed to the loss of biodiversity and deforestation for pasture expansion and feed crop production [126]. The loss of forests and wildlife habitats has increased human exposure to wildlife harbouring microbes that can become zoonotic [127]. The re-emergence of Chagas disease in Brazil, has been attributed to low mammal diversity with similar effects observed for leishmaniasis, Rocky Mountain spotted fever, and schistosomiasis [128].

Neo-tropical animal production has the potential to decrease the emergence and re-emergence of zoonotic diseases, as these species often require reduced land space and utilize local feed resources, which will decrease the negative impact that animal production has on the environment [83, 129]. Additionally, rearing these animals in captivity allows for treatment and vaccination, reducing their microbial load and likelihood of serving as disease vectors.

### Climate Change and neo-tropical species

The survivability of an animal frequently relies on its ability to cope with or adapt to the prevailing climatic conditions. Animals that can modify their phenotypic and physiological traits in response to changing environments are more likely to sustain production amidst the variability and challenges of climate change in the LAC region. Consequently, animals used for production in these regions must be genetically resilient and capable of adapting to the diversity of the evolving environments. Local neo-tropical species possess this ability, being already adapted to the diversity of the environments they naturally inhabit, displaying the natural resilience required for long term production and health.

Local tropical breeds have been identified as having higher thermal comfort zones, enabling them to better cope with the adversities of climate change. This adaptability will likely also be the case for neo-tropical species, which have also demonstrated resilience to heat stress and drought conditions. Supporting this perspective, research that examined the impacts of climate change on livestock in developing countries suggested that the tropics and sub- tropics contained a wealth of animal genetic resources that can be tapped into to assist with heat stress related issues [45]. Moreover, considerable value can be obtained by matching the genetic traits with the physical, biological and economic landscape. Utilising inherent variations in heat tolerance among different species, as identified by researchers, could serve as a crucial strategy for adaptation [11].

In South America the rearing of endemic, wildlife species is an established practice. Since the early 2000’s, countries such as Argentina and Brazil, have been rearing capybaras and collared peccaries in rural areas. The adoption and use of these neo-tropical animals in these regions stem from several factors. Captive rearing provides an easier and more sustainable alternative to wild harvesting, producing higher quality products - particularly important for the leather trade. Furthermore, it helps to meet the demand for wild meat, while reducing the hunting pressure on the wild populations [40].

Neo-tropical avian species, such as the cocrico (*Ortalis ruficauda*), have similarly been reared in Caribbean countries like Tobago [123]. Wildlife farmers have been documented as captive breeding these birds along with the other neo-tropical species like iguanas, agouti and collared peccaries [123, 129]. The cocrico, an endemic bird, is notable for its wide diet and its preference for feeding on local fruits vegetable and seed crops, which has led to its being viewed as a pest species. These features along with its adaptability to the climatic conditions of the LAC region, has led to its recognition as a species with potential for sustainable production in the region [129].

The production of neo-tropical species offers an environmentally friendly alternative to conventional livestock production systems, mitigating the negative impacts that animal production and agriculture have on forests. These species are often smaller in size, and do not require extensive deforestation or the cultivation of special feed crops, as they are already adapted to existing local resources [129]. The reduced pressure on the environment will serve to mitigate the impacts of climate change, which is closely linked to deforestation and ecosystem disruption caused by agriculture and animal production [24, 126].

Researchers emphasize several traits that make neo-tropical species well-suited for captive production, including their environmental adaptability, tolerance to parasites and diseases, and overall hardiness [24, 40]. These traits enable them to thrive under the challenging climatic conditions of the neo-tropics. Neo-tropical wildlife production therefore, has the potential to be an untapped resource for the LAC, and can contribute to improving food security through the production of more climate resilient species and production methods.

### Benefits of neo-tropical animal production

Overall, the ownership of animals and livestock has been recognized as having a number of benefits ranging from improved farmer resilience to better nutrition. The following are the many areas supporting the use of Wildlife farming and neo-tropical animal production.

1. Farmer resilience and human diet diversity

Unconventional livestock species are important genetic resources that can significantly bolster the economies of developing countries [30] by enhancing farmers’ resilience and food security, especially during times of climate change and extreme weather events [74].

This is more prevalent in LAC where small holder farming systems predominate, and livestock are known to play a number of important roles beyond food production [13, 75]. The adoption of diversified livestock systems including non-domesticated animal species has been identified as increasing the diversity of human diets and therefore improving the overall food security of tropical regions [13, 26, 74, 76–79]. Research has already identified that using better adapted animals can be a good strategy and this can extend to wildlife species [79]. Finally for areas that are unable to support traditional agricultural production, wildlife production provides necessary food and income [25, 80–83].

2. Hunting pressure reduction/Conservation

Uncontrolled subsistence hunting has long been identified as unsustainable, leading to drastic declines and extirpations of numerous wildlife populations in the South American region [84]. Evidence supporting the sustainable use of neo-tropical wildlife has been illustrated with studies on caimans in South America, where hunting pressure on wild populations decreased as market demands were satisfied by affordable, captive-bred alternatives [85].

The advantages to the use of non-domesticated species for production in the neo-tropics include several conservation and research benefits, These include their role in conservation by serving as insurance populations for wild counterparts that may be overhunted or under threat by natural events such as diseases [130]. In addition, captive production of neo-tropical species will provide researchers with increased opportunities to collect valuable biological and physiological data on these species, which can then be used to protect their wild populations [129]. These species can further be used in educational programmes to raise awareness and share knowledge about understudied neo-tropical animals [24, 129]. Finally, the use of captive bred neo-tropical animals can help to reduce hunting pressure on wild populations [24, 25, 80–83].

This approach highlights the potential of non-domesticated, neo-tropical species to support both conservation and sustainable production, while further addressing the challenges posed by overhunting in the LAC.

3. Better adaptation to heat stress.

It has already been found in recent research that more tropical-adapted breeds are less susceptible to the effects of heat stress, with the dairy cow breeds that fall into *Bos indicus* having been noted as being better adapted to tropical environments [12]. This concept can be applied to neo-tropical and tropical wildlife species, which, like tropical livestock breeds, are naturally adapted to the heat and humidity of the region. The Collared peccary for example, naturally occupies a wide range of habitats ranging from arid to tropical environments, illustrating its adaptability to the environment and climates in the neo-tropics [86].

Similarly, the capybara is recognized for its ability to tolerate heat and resist local diseases and parasites [25]. This assumption can be strengthened with further research, as evidenced by the management of wildlife species in Africa. In these regions, endemic game animals including various species of antelope and rodents, naturally adapted to harsh conditions, are bred and utilized on game ranches for economic purposes [79, 124].

4. Reduced pollution.

Livestock production significantly contributes to climate change, primarily through greenhouse gas emissions [90], substantial carbon and water footprints [78] and water pollution [90]. Traditional livestock species, in particular, are major greenhouse gases (GHG) emitters [90], and account for approximately 70% of freshwater use [45]. Wildlife production and utilizing endemic neo-tropical species has the advantage of utilizing locally available feeds [24], having diverse diets [26, 91] and having reduced drinking water requirements being already adapted to neo-tropical environments.

5. Economic benefits.

Captive rearing of endemic wildlife is often identified by wildlife farmers as providing an alternative source income. Initial studies on the economic costs of breeding both the capybara and the collared peccary, as compared to domesticated pig breeding, found that the animal net income was lower than that of the domesticated pig, due to the higher production costs [40]. This might be mitigated by the higher cost of peccary and capybara meat, along with the use of improved breeding techniques, to increase production and selective breeding for genetic improvement, thereby supporting the use of these animals economically as an alternative source of protein and income for farmers for the region [40].

6. Nutrition and other uses.

For species like the peccary and capybara, the meat has been analysed and found to be much lower in fat and cholesterol resulting in a healthier meat that consumers prefer in South America, as compared to the fattier domestic versions [92]. In addition to serving as an important meat protein alternative, some wildlife species are valued for their hides and medicinal properties. For example, capybara leather, used to make luxury items, has a ready international market from Europe, USA and Japan [40]. Similarly, the hide of the collared peccary has been identified as a suitable material for leather production.

### Challenges and limitations

The use of non-traditional wildlife for farming has been adopted in certain regions and while many have shown promise, certain setbacks and challenges have been identified.

Some of the concerns raised include that wildlife farming can harm native wildlife population, and that it has a high cost of production versus subsistence hunting, and further that cultural constraints would hinder this type of activity [93, 94]. However, many studies have already highlighted that excessive harvesting, including subsistence hunting, can lead to negative impacts on wildlife [79], and in many countries in the LAC regions, wild meat consumption is practised widely and wildlife farming is culturally accepted [95, 96]. Finally, and perhaps most importantly, the production of non-conventional animals is an age-old practice that predates domestic animal production [82].

Regulation compliance with conservation laws and agricultural regulations must be considered. In the case of the LAC region, the laws have not yet been updated to reflect the position of wildlife as farmed species, and thus do not serve to safeguard or support captive rearing of wildlife other than for research [25]. In Trinidad and Tobago, the Conservation of Wildlife Act is restricted mainly to protecting certain game and listed species, preventing their captive rearing and sale during hunting seasons [97]. An additional setback is the national status of the species, if recognized as a national symbol like the cocrico in Trinidad and Tobago, hunting and production use would be prohibited [123]. In the greater LAC, many laws were found to not effectively address the needs of conservation, production, and utilization of neo-tropical animal species, with a further lack of political and educational priorities geared specifically towards neo-tropical animal education, use, conservation, and production [98].

For example, the collared peccary was placed on the Convention on the International Trade in Endangered Species of Wild Fauna and Flora (CITES) Appendix II in 1986. This required a CITES permit for international trade and certification of the breeding facility by CITES. Although the listing increased the regulations and procedures involved with rearing and trading the species, it also serves as a means to monitor its trade and reduce any illegal use of the animals [99]. However, Brazil recently instituted nine categories of wildlife for use and management in captivity with a number of neo-tropical animal breeding sites registered with the Brazilian Institute of Environment and Renewable Resources (IBAMA) (IBAMA No 169 2008) from [100, 101], and as of 2011 Le Pendu et al. [101] reported a number of commercial breeding sites for mammals, birds and reptiles including capybara, paca, agouti, rhea (*Rhea americana*) and *Caiman yacare.*

Animal welfare concerns, abuse, and overexploitation of wild populations have been identified in some Asian wildlife farming practices, where wild populations are often used as seed stock, exemplified with the dramatic decline of the porcupine (*Hystrix brachyura*) [102]. Contrastingly, such issues have not been identified in the Caribbean region. Wildlife farming in the Caribbean, as practiced by local farmers, has not been found to be unsustainable or negatively impacting wild populations [123, 129]. However, given that wildlife farming in these regions is practised on smaller scale, there are few studies that have explored its long-term impact. Thus greater study is required to understand its effect fully in the LAC.

Another significant concern is the illegal laundering of wild-caught animals as captive stock. This issue has been identified with the Collared peccary, a species actively being produced in South America [103]. The reduction of the inhumane, unethical practices requires proper monitoring systems to be established. These systems should include registering and identifying captive animals using ear tags or microchips - a practice already established in domesticated cattle in Brazil [24].

Regarding the sourcing of founder populations for wildlife farming, alternative approaches can reduce the risk of overexploitation These include obtaining founder populations for certain neo-tropical wildlife species from areas where the species are considered an agricultural pest or via the establishment of captive breeding centres by NGO’s or government agencies to supply founder stock [24]. These methods would help to minimize the pressure on wild populations while supporting sustainable wildlife farming.

Wildlife farming has received a bad reputation for housing animals in conditions that are not humane or comfortable [104]. This however can be mediated by greater research, and developing production systems that are humane and built with the animal’s behaviour and biology in mind, along with the use of appropriate husbandry practices that allow for improved productivity and reduced costs [105]. Support for this can be found with collared peccary studies [83, 106]. These studies all highlighted that semi-intensive production of collared peccaries resulted in increased production as compared to intensive systems, likely due to the more humane conditions the animals were under.

A potential concern in wildlife farming is the issue of escapees, or escaped animals establishing invasive populations. This issue can be mitigated by wildlife farmers focussing on endemic species already native to the country. Many of the species proposed for production, such as the capybara and collared peccary, are naturally found in Latin America, while species like the agouti, iguana and caiman have natural ranges that include Latin America and the Caribbean. Since these species are not domesticated, escapees will be able to reintegrate into their native wild populations without disrupting the local ecosystems.

Finally, some authors recognize that wildlife animals are not domesticated and therefore are not adapted to being held in captivity, thus the innate and social behaviour of the animal is important and needs to be factored into the production systems [27] to ensure that the animal is held under humane conditions. It is important to consider that wildlife are sentient species and therefore it might prove beneficial for producers of wildlife species to consider use of an ethical framework to assess the suitability of certain species for production [107]. Generally, however, information on the behaviour of many neo-tropical species is sparse and much greater research is needed on neo-tropical wildlife species [27].

Limited information is available on the numbers of wildlife being farmed [96, 108], the biology of these animals, and on effective captive rearing of wildlife species in the region [101]. Research into captive breeding requirements, nutrition, and disease management for these species is often limited [24, 74]. This makes it difficult for new farmers to develop efficient systems for reliable production, and without a good understanding of their specific needs, large-scale production could negatively impact their well-being. Nonetheless, great strides have been made in the LAC with recent research being done on the utilization and production of neo-tropical species with potential for production including the agouti, paca, caiman and opossum [34, 83, 87–89, 109].

Concerns exist over animal health and zoonotic spread of disease through wildlife farms [110]. The capybara is often cited as a host of ticks that transmit spotted fever [111, 112]. The rodent, *Agouti paca*, can harbour leishmaniasis and trypanosomiasis, leading to human diseases [113]. These risks can be greatly reduced once biosecurity protocols are in place, such as the species being captive reared having no contact with wildlife; the species being treated using the correct disease management health protocols specific to neo-tropical species; practicing quarantine biosecurity systems to reduce the animals carrying or hosting parasites; and being reared in healthy sanitary conditions that do not foster infections and spread of diseases [24]. Although this issue is a serious one, the alternative of continued use of wild-caught populations for subsistence and commercial use presents a much greater risk of zoonotic spread, with African studies identifying that bushmeat that is hunted, transported, handled and cooked in an unsafe manner can lead to detrimental health impacts [114].

Non-traditional species may face challenges in market acceptance. Due to unfamiliarity among consumers, potential cultural preferences for traditional livestock and classification differences for wildlife all have a remarkable impact on their acceptability for consumption [115]. Despite these concerns, there are still populations that heavily rely on and utilize wildlife in the LAC. For example, in Brazil, the meat of wild animals is well accepted by the population [101] and people have reported a high consumption of meat from species that include paca and armadillo (Genus *Euphractus)* [116]. Further support for wildmeat acceptance in the market can be found in the Caribbean, where wild meat is a popular meat being consumed by the population and there being an interest and established practice of wildlife farming [117, 123].

Scarcity of data on the nutritional content of most common wildlife species has led people to undervalue wild meat. There are still those who are under the misconception that ‘wild’ or ‘bush’ meat is of an inferior quality or nutritive value than that of livestock meat species [26]. It must be noted that available studies in Africa demonstrate that bushmeat/wild meat is an important source of fats, micro and macro-nutrients and has a diversity of medicinal uses [114]. Recent studies in LAC have also made great strides in researching and providing important nutritional facts on wild species like the caiman, capybara [34, 118] agouti and collared peccary [28, 119]. However much greater research is required on the nutritional content in the form of comparative nutritional studies to livestock species to inform and influence people toward wild meat use and consumption.

There is a lack of established infrastructure and expertise to ensure a reliable supply chain [25]. Research has reported that some of the difficulties faced by wildlife farmers ranged from lack of technical assistance and financial support, bureaucracy and high production costs [120]. However, since that time much research has indicated that wildlife production can be conducted and prove effective [40, 83, 106].

With many high-producing domesticated, animals unable to produce at high capacity, due to the counteracting effects of the climate and environment, often succumbing to the effects of heat stress or pathogens, tropical animals have been found to be very viable and productive and ecomonically profitable [121, 125]. As shown with guinea pigs (*Cavia porcelus*) [24], genetic selection programmes can greatly increase reproduction and thus raise reproduction rates [122].

## Conclusions

The above paper provides some information and solutions on animal production in the tropics within an ever-changing climate. It was demonstrated that conventional, domesticated animals may be less suitable for animal production in the LAC region due to increased energy and protein requirements under heat stress, which reduces production, reproduction, immunity and survival while increasing mortality and morbidity. An alternative is the utilization and production of smaller non-domesticated, neo-tropical animal species. These animals are well adapted to the environmental conditions in the region and can survive and be productive on locally acquired feedstuff. There are however some constraints in the use of these animals such as the lack of technical expertise and government support through subsidies. Future work must be conducted in the improvement of laws and governmental support in rearing these wildlife species for commercial use. While greater research focus is needed on obtaining knowledge on neo-tropical species reproductive parameters and nutritional needs in captivity to aide in captive management, along with the welfare and economic impact of climate change on agriculture in the LAC.

## Data Availability

Data sharing is not applicable to this article as no datasets were generated or analysed during the current study.
